# Supplementary material for: Live bearing promotes the evolution of sociality in reptiles
Source: Nat Commun. 2017 Dec 11;8:2030. doi: 10.1038/s41467-017-02220-w (PMC5725568; doi:10.1038/s41467-017-02220-w)
Supplement: Supplementary file 1 — Supplementary Information [file 41467_2017_2220_MOESM1_ESM.pdf]

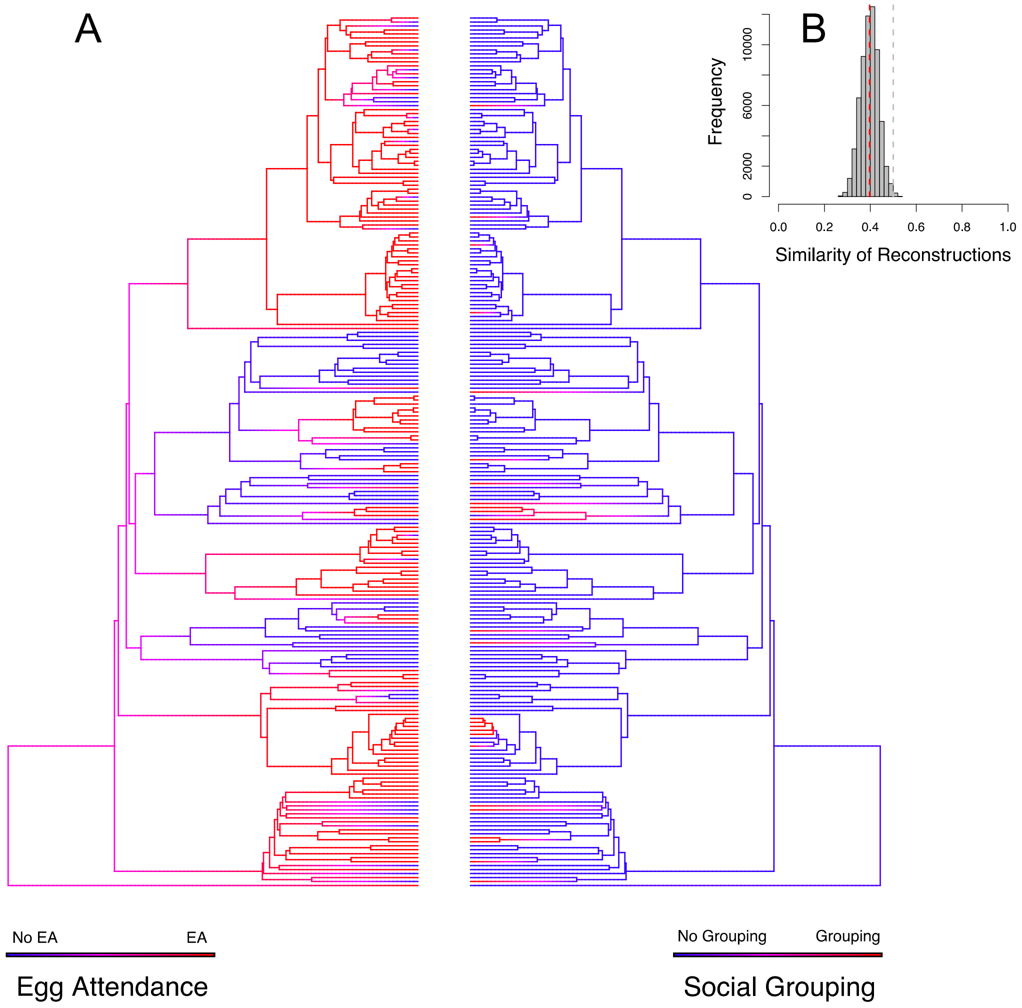

Supplementary Figure 1. No evidence for correlated evolution between egg attendance and social grouping among oviparous squamate reptiles . (a) Ancestral state reconstructions of egg attendance and social grouping by stochastic character mapping. Phylogeny restricted to oviparous species in the conservative data set (n = 219, see below). Branch colours represent posterior probability densities of edge states based on 1000 stochastic character maps of each reconstruction. (b) Distribution of similarity scores between stochastic character map sets (n = 1000) based on separate ancestral character state reconstructions of egg attendance and social grouping. The grey line represents the null expectation of similarity between map sets if the two traits being compared show no evolutionarily correlation during reconstruction. The red line represents the mean similarity between map sets based on our reconstructions.

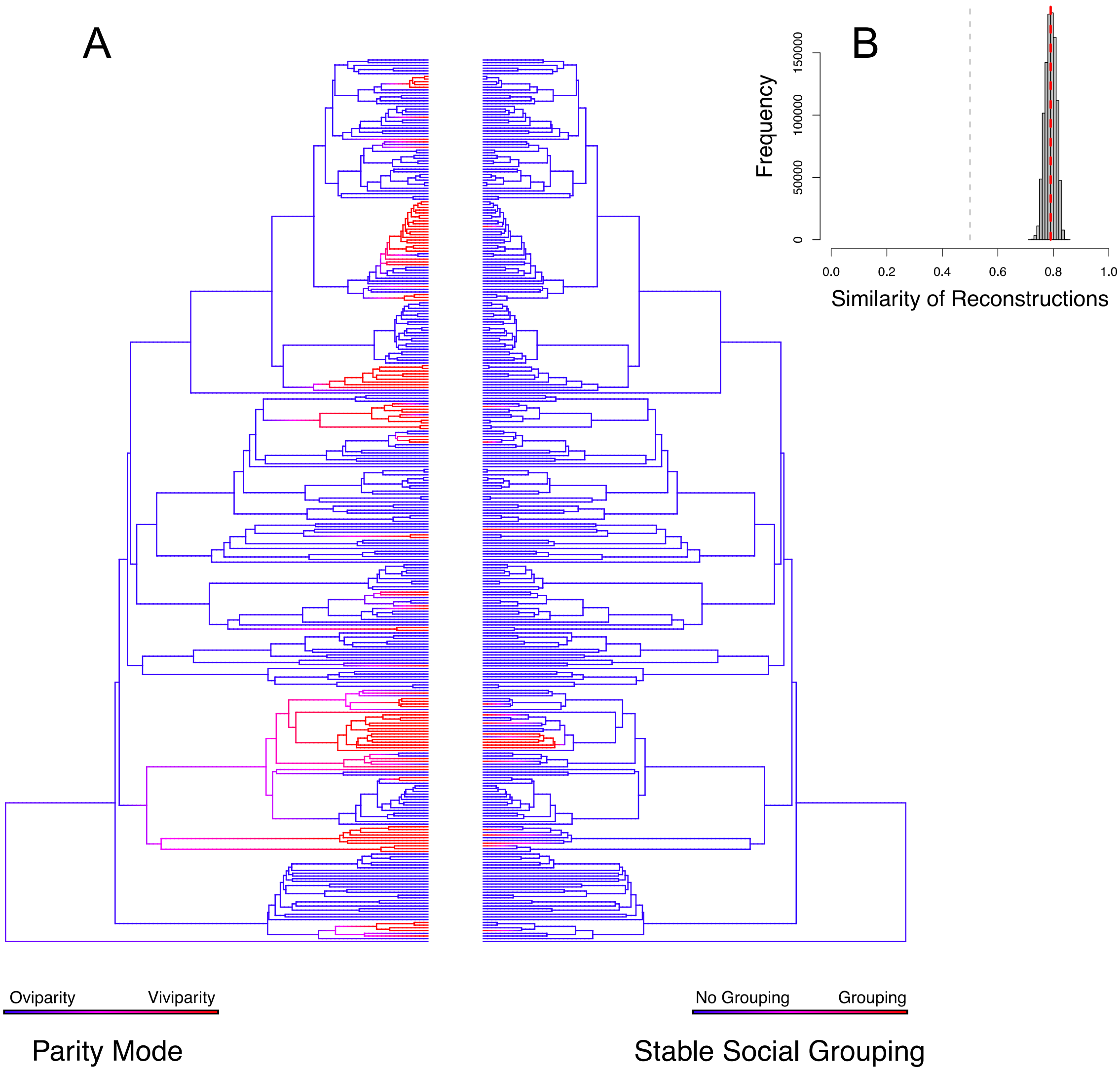

Supplementary Figure 2. Correlated evolution of viviparity and stable social grouping among squamate reptiles. (a) Ancestral state reconstructions of parity mode and stable social grouping by stochastic character mapping. Phylogeny restricted to species in the conservative data set (n = 324, see below). Branch colours represent posterior probability densities of edge states based on 1000 stochastic character maps of each reconstruction. (b) Distribution of similarity scores between stochastic character map sets (n = 1000) based on separate ancestral character state reconstructions of egg attendance and social grouping. The grey line represents the null expectation of similarity between map sets if the two traits being compared show no evolutionarily correlation during reconstruction. The red line represents the mean similarity between map sets based on our reconstructions.

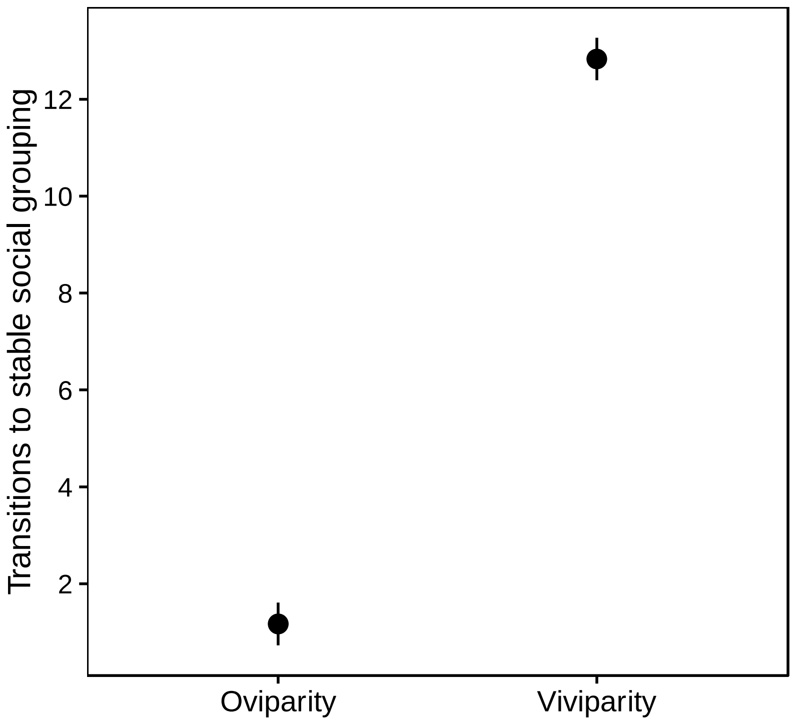

Supplementary Figure 3. Number of independent transitions to stable social grouping from viviparity and oviparity. Dots represent the mean (± one standard deviation) number of transitions from a background of each parity mode based on 1000 stochastic character maps of the joint reconstruction of parity mode and stable social grouping.

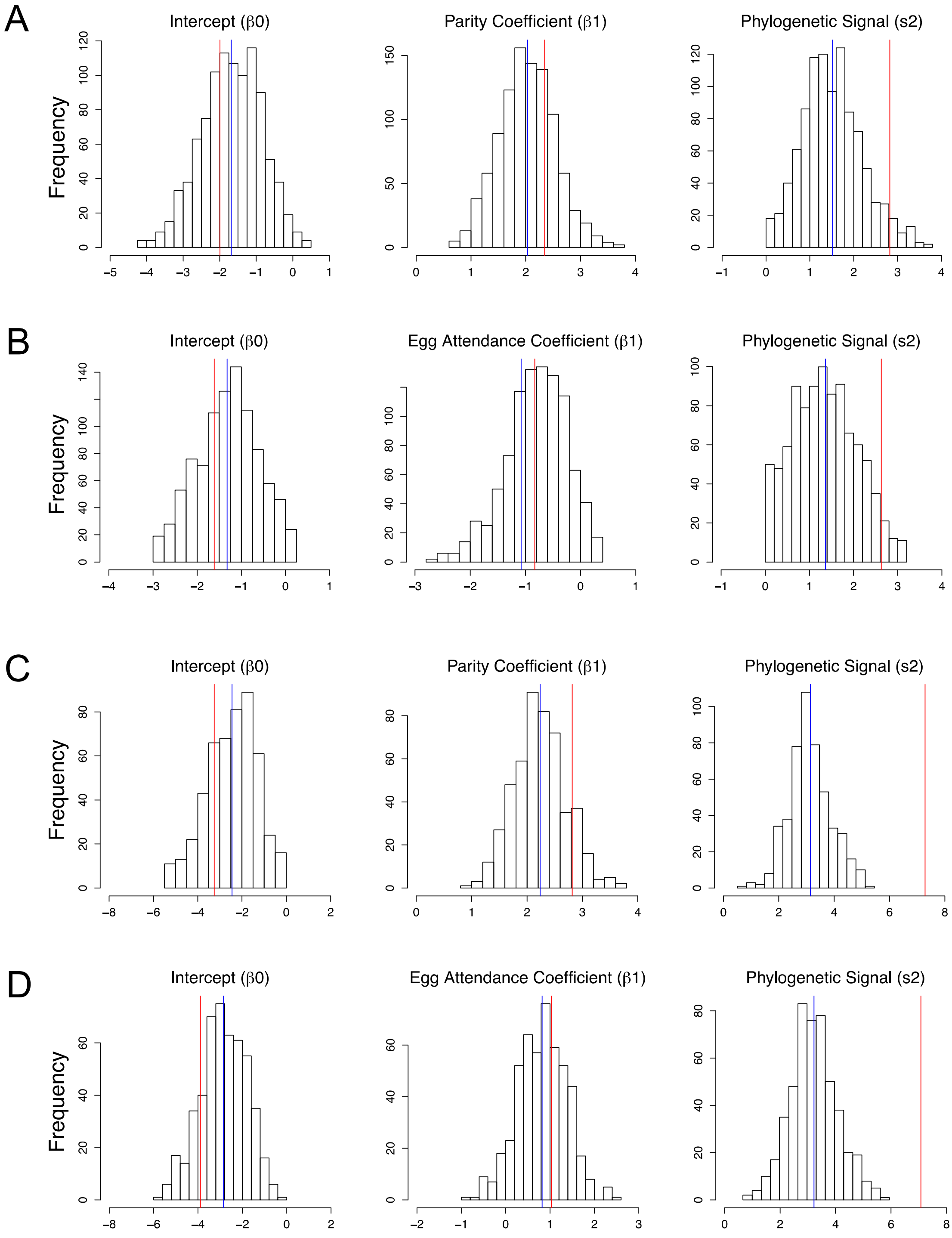

Supplementary Figure 4. Posterior distributions of parameter estimates (β0, β1, s2) from binary phylogenetic generalized linear mixed models of the relationship between parity mode and social grouping in squamate reptiles from simulated data. The blue lines represent the mean of the posterior distribution of each parameter estimate from 1000 (a, b) or 500 (c, d) model simulations. The red lines represent the original parameter estimates from the real data models (Table 1). (a) Conservative zeros data set, parity model. (b) Conservative zeros data set, egg attendance model. (c) Relaxed zeros data set, parity model. (d) Relaxed zeros data set, egg attendance model. Estimates at the extreme of each distribution (falling below the 0.5^{th} and above the 99.5^{th} percentiles) are excluded.

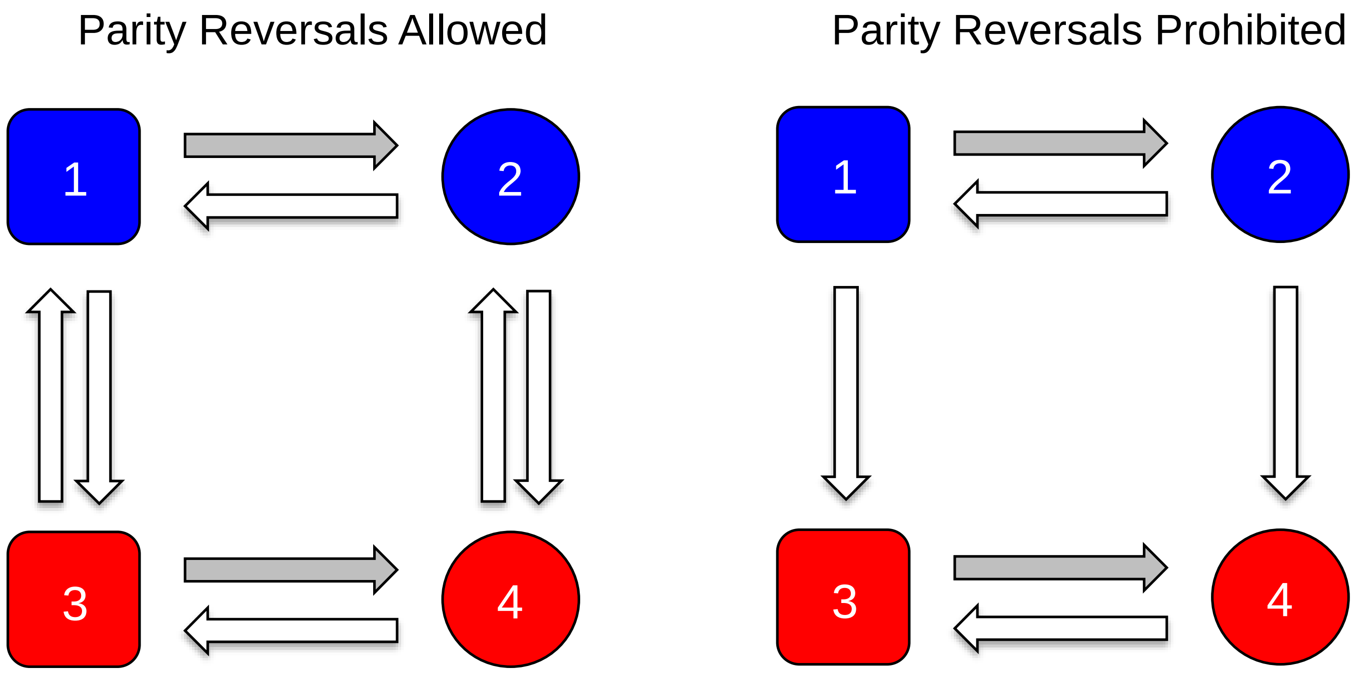

Supplementary Figure 5. Structure of the two MuSSE models used to estimate rates of transition toward social grouping (SG) from a background of each parity mode. Blue represents oviparity, red represents viviparity; Squares are social grouping absent, circles are social grouping present. Simultaneous double transitions are prohibited (i.e. no diagonal arrows). Numbers correspond to the four possible states in each model (1: oviparous, no SG; 2: oviparous, SG; 3: viviparous, no SG; 4: viviparous, SG; also see Supplementary Table 3). Grey arrows represent the transition rates that were constrained to be equal during likelihood ratio tests of the difference in transition rate towards SG between parity modes (i.e. 'Test' models in Supplementary Table 3).

Supplementary Table 1. **Number of species from different squamate families displaying each form of social grouping.** Numbers in parentheses represent the subset of all species within a category that are included in the phylogeny of ref. [1]. Where no parentheses are shown all species are included in the phylogeny. Includes species reported as displaying both oviparity and viviparity (excluded from analyses).

	Social Grouping	Stable Social Grouping
Amphisbaenia		
Trogonophidae	1	0
Amphisbaenidae	1	0
**Total**	**2**	**0**
Sauria		
Agamidae	7	1
Anguidae	2 (1)	0
Carphodactylidae	1	0
Cordylidae	6	3
Diplodactylidae	4	1
Geckkonidae	4	0
Lacertidae	2	0
Leiocephalidae	1	0
Liolaemidae	6 (5)	1
Phrynosomatidae	1	1
Phyllodactylidae	1	0
Scincidae	30 (25)	12 (9)
Tropiduridae	1	0
Xantusiidae	2	1
Xenosauridae	3 (1)	0
**Total**	**70 (61)**	**20 (17)**
Serpentes		
Boidae	1	0
Colubridae	1	0
Elapidae	1	0
Pythonidae	2	0
Typhlopidae	1 (0)	0
Viperidae	17	1
**Total**	**23 (22)**	**1**
**Grand Total**	**96 (86)**	**21 (18)**

Supplementary Table 2. Number of species from different squamate families displaying each parental care trait listed in Supplementary Table 4. Many species display multiple forms of care, therefore the final column shows the total number of species from each family reported to show any form of care. Numbers in parentheses represent the subset of all species within a category that are included in the phylogeny of ref. [1]. Where no parentheses are shown all species are included in the phylogeny. Includes species reported as displaying both oviparity and viviparity (excluded from analyses).

	Nesting	Egg Manipulation	Defence	Neonatal Assistance	Parent-offspring Association	Number of Species
**Amphisbaenia**						
Trogonophidae	0	0	0	0	1	1
Amphisbaenidae	0	0	0	0	1	1
**Total**	**0**	**0**	**0**	**0**	**2**	**2**
**Sauria**						
Agamidae	7	0	5 (4)	1	6	12 (11)
Anguidae	3	14 (8)	5 (3)	3	2 (1)	19 (12)
Cordylidae	1	0	0	1	3	3
Crotaphytidae	0	0	1	0	0	1
Dactyloidae	2	0	0	0	0	2
Diplodactylidae	0	0	3	0	3	4
Gekkonidae	2	10	11	2	2	14
Iguanidae	12	0	12	0	0	14
Lacertidae	2	2	1	0	2	5
Leiocephalidae	0	0	0	0	1	1
Liolaemidae	1	0	2	1	6 (5)	7 (6)
Phrynosomatidae	3	0	0	0	1	4
Phyllodactylidae	4	3	3	0	1	5
Scincidae	18 (15)	27 (23)	17 (13)	12 (10)	27 (23)	54 (44)
Sphaerodactylidae	1	0	1	0	0	2
Teiidae	2	2	2	0	0	3
Tropiduridae	3	0	3	0	1	4
Varanidae	9	1	6	2	0	13
Xantusiidae	0	0	0	2	2	3
Xenosauridae	0	0	0	0	3 (1)	3 (1)
**Total**	**70 (67)**	**59 (49)**	**72 (65)**	**24 (22)**	**60 (52)**	**173 (152)**
**Serpentes**						
Boidae	0	9	2	4	1	9
Calabariidae	0	1	0	0	0	1
Colubridae	8 (7)	20 (18)	5	0	0	23 (21)
Elapidae	5	17 (14)	8 (7)	0	0	17 (14)
Lamprophiidae	1	4 (3)	0	0	0	4 (3)
Leptotyphlopidae	1	1	0	0	0	1
Pythonidae	4	27 (23)	10 (8)	0	2	27 (23)
Viperidae	1	14 (12)	15 (14)	0	16	28 (26)
**Total**	**20 (19)**	**93 (81)**	**40 (36)**	**4**	**19**	**110 (98)**
**Grand Total**	**90 (86)**	**152 (130)**	**112 (101)**	**28 (26)**	**81 (73)**	**285 (252)**

Supplementary Table 3. Parameter estimates and likelihoods for alternative models constructed in the MuSSE framework. State dependent speciation (**λ**) and extinction (**μ**) rates are allowed for all states (1: oviparous, no SG; 2: oviparous, SG; 3: viviparous, no SG; 4: viviparous, SG). Light grey cells indicate transition rates that have been fixed to be equal. Black cells indicate transition rates that have been constrained to zero (parameters representing simultaneous double transitions (e.g. q14) constrained to zero but not shown). In test models, rates of transition toward social grouping from a background of oviparity and viviparity have been constrained to be equal to perform a likelihood ratio test comparing differences in model fit when transition rates toward social grouping from each parity mode are allowed to vary. Transition rates toward social grouping are always higher from a background of viviparity (q34) than oviparity (q12) whether parity reversal are allowed or prohibited. Significant P values in bold.

Parity reversals	Model	Df	ΔAIC	X^2	P	λ1	λ2	λ3	λ4	μ1	μ2	μ3	μ4	q12	q13	q21	q24	q31	q34	q42	q43
**Conservative zeros data set, complete cases analysis (n = 324)**																					
Prohibited	Fit	14				0.422	0.055	0.872	0.008	0.362	<0.001	0.746	<0.001	0.002	0.006	0.028	<0.001		0.116		0.024
	Test	13	31.1	33.1	**<0.001**	0.425	0.052	0.610	0.383	0.360	<0.001	0.593	0.282	0.004	0.009	0.027	0.002		0.004		0.020
Allowed	Fit	16				0.419	0.054	0.874	0.008	0.358	<0.001	0.749	<0.001	0.002	0.006	0.028	<0.001	<0.001	0.116	<0.001	0.024
	Test	15	14.5	16.5	**<0.001**	0.506	0.049	0.503	0.418	0.463	<0.001	0.446	0.333	0.005	0.002	0.033	0.003	0.020	0.005	<0.001	0.015
**Relaxed zeros data set, complete cases analysis (n = 1210)**																					
Prohibited	Fit	14				0.155	0.035	0.214	0.116	0.064	<0.001	0.132	0.045	0.001	0.002	0.014	0.002		0.010		0.011
	Test	13	12	14.2	**<0.001**	0.152	0.037	0.264	0.126	0.066	<0.001	0.201	0.046	0.001	0.002	0.015	0.004		0.001		0.017
Allowed	Fit	16				0.152	0.034	0.319	0.112	0.070	<0.001	0.259	0.045	0.001	<0.001	0.011	0.004	0.022	0.002	0.000	0.028
	Test	15	19	21.1	**<0.001**	0.144	0.039	0.399	0.124	0.056	<0.001	0.355	0.084	0.001	<0.001	0.017	0.002	0.015	0.001	0.002	0.015
**Conservative zeros data set, all species in phylogeny (n = 3951, response of species with unknown state of social grouping (n = 3627) estimated by model)**																					
Prohibited	Fit	14				0.094	0.024	0.204	0.014	<0.001	<0.001	<0.001	0.010	0.033	0.009	0.010	<0.001		0.171		0.001
	Test	13	154	156.3	**<0.001**	0.122	0.033	0.009	0.074	<0.001	<0.001	<0.001	<0.001	0.017	0.043	0.010	<0.001		0.017		0.044
Allowed	Fit	16				0.062	<0.001	0.209	<0.001	<0.001	<0.001	<0.001	0.043	0.029	0.004	0.001	0.040	0.075	0.100	<0.001	0.001
	Test	15	21	22.3	**<0.001**	0.063	<0.001	0.191	<0.001	<0.001	0.003	<0.001	0.042	0.022	0.004	<0.001	0.083	0.115	0.042	<0.001	0.001
**Relaxed zeros data set, all species in phylogeny (n = 3951, response of species with unknown state of social grouping (n = 2741) estimated by model)**																					
Prohibited	Fit	14				0.074	0.051	0.170	0.011	<0.001	<0.001	<0.001	<0.001	<0.001	0.002	0.007	0.010		0.091		0.004
	Test	13	147	149.2	**<0.001**	0.076	0.042	0.152	0.078	<0.001	<0.001	<0.001	<0.001	0.001	0.001	0.006	0.001		0.001		0.005
Allowed	Fit	16				0.060	0.088	0.171	0.011	<0.001	<0.001	<0.001	<0.001	<0.001	<0.001	0.009	0.065	0.065	0.035	0.012	0.004
	Test	15	35	37.7	**<0.001**	0.050	0.014	0.177	0.096	<0.001	<0.001	<0.001	<0.001	0.001	0.001	0.004	0.012	0.128	0.001	0.055	0.016

Supplementary Table 4. **Phylogenetic generalized linear mixed models (PGLMM) testing the influence of parity mode and egg attendance on the occurrence of social grouping in squamate reptiles.**

	Data Set	Parameter	Estimate	Test Statistic	Confidence Interval 2.5%	Confidence Interval 97.5%	Bias
**Parity Mode**							
	Conservative (n = 324)	Intercept (β0)	-2.01 ± 0.96	**Z = -2.09, P = 0.04**	-3.45	-0.14	-0.31
		Parity Mode (β1)	2.34 ± 0.42	**Z = 5.59, P < 0.001**	1.02	3.09	0.31
		Citation Count	<0.001 ± 0.001	Z = 0.27, P = 0.79	-	-	-
		Signal in Residuals (s2)	2.84	**P < 0.001**	0.23	3.11	1.31
		Signal in Response (s2)	4.19	**P < 0.001**	-	-	-
	Relaxed (n = 1210)	Intercept (β0)	-3.25±1.50	**Z = -2.16, P = 0.03**	-5.03	-0.42	-0.80
		Parity Mode (β1)	2.82±0.49	**Z = 5.70, P < 0.001**	1.30	3.31	0.58
		Signal in Residuals (s2)	7.28	**P < 0.001**	1.73	4.81	4.14
		Signal in Response (s2)	8.92	**P < 0.001**	-	-	-
**Egg Attendance (analyses restricted to oviparous species)**							
	Conservative (n = 219)	Intercept (β0)	-1.61 ± 0.99	Z = 1.62, P = 0.11	-2.98	0.24	-0.30
		Egg Attendance (β1)	-0.83 ± 0.54	Z = -1.52, P = 0.13	-2.71	0.28	-0.25
		Citation Count	<0.001 ± 0.001	Z = -0.06, P = 0.95	-	-	-
		Signal in Residuals (s2)	2.66	**P < 0.001**	<0.001	3.15	1.27
		Signal in Response (s2)	2.35	**P < 0.001**	-	-	-
	Relaxed (n = 1049)	Intercept (β0)	-3.89 ± 1.51	**Z = 2.58, P = 0.01**	-5.14	-0.89	-1.03
		Egg Attendance (β1)	1.04 ± 0.63	Z = 1.66, P = 0.10	-0.42	1.92	0.21
		Signal in Residuals (s2)	7.07	**P < 0.001**	1.50	5.11	3.86
		Signal in Response (s2)	7.92	**P < 0.001**	-	-	-

N equals the number of species included in each analysis. The 'signal in response (s2)' parameter is derived from a model fit with no predictor variables and provides an estimate of phylogenetic structure of social grouping from each data set. Model estimates are reported ±SE. Confidence intervals of parameter estimates by parametric bootstrapping are derived from PGLMMs performed on simulated data (1000 and 500 simulations of the conservative and relaxed data sets, respectively; see Methods for details). Bias values represent the difference between parameter estimates from each real data model and the mean of the posterior of parameter estimates from simulations. Significant terms are shown in bold.

Supplementary Table 5. Definitions of parental care behavior used to categories reports of care into different functional modes.

Care Mode	Definition
Nesting Behaviour	Construction of a nest space in which to receive eggs. This includes species that excavate nests and those that construct nests from leaf litter and debris. Does not include communal nesting.
Egg Manipulation	Any behavior involving parental contact or manipulation of the eggs. This includes brooding behavior, relocation of eggs during incubation and removal or consumption of non-viable eggs.
Defense	Any behavior in which parents are observed to respond defensively to potential predators approaching the eggs or nest.
Neonatal Assistance	Reports of parents assisting hatchlings/neonates out of their shell/birthing membrane during hatching/parturition.
Parent-Offspring Association	Sustained association or tolerance between parent(s) and offspring after hatching or birth. Includes active or passive defense of neonates, crevice or burrow sharing, and postponed dispersal in which offspring remain within the parental territory beyond the birthing/hatching period.

Supplementary Table 6. Parameter estimates and likelihoods for MuSSE models exploring the alternative causal explanation that social grouping promotes the evolution of viviparity. State dependent speciation (**λ**) and extinction (**μ**) rates are allowed for all states (1: oviparous, no SG; 2: oviparous, SG; 3: viviparous, no SG; 4: viviparous, SG). Light grey cells indicate transition rates that have been fixed to be equal. Black cells indicate transition rates that have been constrained to zero (parameters representing simultaneous double transitions (e.g. q14) constrained to zero but not shown). In test models, rates of transition toward viviparity from a background of social grouping and no social grouping have been constrained to be equal to perform a likelihood ratio test comparing differences in model fit when transition rates toward viviparity from each mode of sociality are allowed to vary. Transition rates toward viviparity from a background of no social grouping (q13) are equal to or higher than from a background of social grouping (q24) in four out of eight models. Furthermore, transition toward social grouping from a background of viviparity (q34) was higher than transitions to viviparity from a background of social grouping (q24) in all but the two least conservative models (relaxed data set, parity reversals allowed). Significant P values in bold. Hyphens indicate test statistics for likelihoods ratio tests that failed to converge.

Parity reversals	Model	Df	ΔAIC	X^2	P	λ1	λ2	λ3	λ4	μ1	μ2	μ3	μ4	q12	q13	q21	q24	q31	q34	q42	q43
**Conservative zeros data set, complete cases analysis (n = 324)**																					
Prohibited	Fit	14				0.422	0.055	0.872	0.008	0.362	<0.001	0.746	<0.001	0.002	0.006	0.028	<0.001		0.116		0.024
	Test	13	3.8	5.79	**0.02**	0.417	0.057	0.748	0.042	0.357	<0.001	0.602	0.049	0.002	0.005	0.002	0.005		0.127		0.016
Allowed	Fit	16				0.419	0.054	0.874	0.008	0.358	<0.001	0.749	<0.001	0.002	0.006	0.028	<0.001	<0.001	0.116	<0.001	0.024
	Test	15	2.2	4.24	**0.04**	0.449	0.058	0.578	0.095	0.396	<0.001	0.430	0.095	0.002	0.003	0.029	0.003	0.005	0.107	<0.001	0.012
**Relaxed zeros data set, complete cases analysis (n = 1210)**																					
Prohibited	Fit	14				0.155	0.035	0.214	0.116	0.064	<0.001	0.132	0.045	0.001	0.002	0.014	0.002		0.010		0.011
	Test	13	-	-	-	0.147	0.035	0.203	0.062	0.060	<0.001	0.082	<0.001	0.001	0.001	0.014	0.001		0.023		0.008
Allowed	Fit	16				0.152	0.034	0.319	0.112	0.070	<0.001	0.259	0.045	0.001	<0.001	0.011	0.004	0.022	0.002	0.000	0.028
	Test	15	3	4.64	**0.03**	0.138	0.042	0.262	0.074	0.045	<0.001	0.181	0.048	<0.001	0.001	0.021	0.001	0.008	0.017	0.005	0.006
**Conservative zeros data set, all species in phylogeny (n = 3951, response of species with unknown state of social grouping (n = 3627) estimated by model)**																					
Prohibited	Fit	14				0.094	0.024	0.204	0.014	<0.001	<0.001	<0.001	0.010	0.033	0.009	0.010	<0.001		0.171		0.001
	Test	13	3	4.94	**0.03**	0.95	0.025	0.212	0.007	<0.001	<0.001	<0.001	0.016	0.038	0.007	0.010	0.007		0.174		0.001
Allowed	Fit	16				0.062	<0.001	0.209	<0.001	<0.001	<0.001	<0.001	0.043	0.029	0.004	0.001	0.040	0.075	0.100	<0.001	0.001
	Test	15	15	16.92	**<0.001**	0.058	0.001	0.188	0.050	<0.001	<0.001	<0.001	<0.001	0.021	0.002	0.006	0.002	0.118	0.023	<0.001	0.010
**Relaxed zeros data set, all species in phylogeny (n = 3951, response of species with unknown state of social grouping (n = 2741) estimated by model)**																					
Prohibited	Fit	14				0.074	0.051	0.170	0.011	<0.001	<0.001	<0.001	<0.001	<0.001	0.002	0.007	0.010		0.091		0.004
	Test	13	13	14.7	**<0.001**	0.074	0.044	0.172	0.013	<0.001	<0.001	<0.001	<0.001	0.001	0.002	0.007	0.002		0.094		0.006
Allowed	Fit	16				0.060	0.088	0.171	0.011	<0.001	<0.001	<0.001	<0.001	<0.001	<0.001	0.009	0.065	0.065	0.035	0.012	0.004
	Test	15	45	37.3	**<0.001**	0.059	0.003	0.171	0.057	<0.001	0.011	<0.001	<0.001	<0.001	<0.001	<0.001	<0.001	0.07	0.038	0.029	0.006
