# Supplementary material for: Live bearing promotes the evolution of sociality in reptiles
Source: Nat Commun. 2017 Dec 11;8:2030. doi: 10.1038/s41467-017-02220-w (PMC5725568; doi:10.1038/s41467-017-02220-w)
Supplement: Supplementary file 3 — Description of Additional Supplementary Files [file 41467_2017_2220_MOESM3_ESM.pdf]

File Name: Supplementary Data 1
Description: This data set contains all the data used to examine the role of parity mode in the evolution of social grouping in squamate reptiles as well as the references to the relevant literature.

File Name: Supplementary Data 2
Description: This data set contains all the data used to examine the role of parity mode in the evolution of stable social grouping in squamate reptiles as well as the references to the relevant literature.
